# Genetic recombination is associated with intrinsic disorder in plant proteomes

**DOI:** 10.1186/1471-2164-14-772

**Published:** 2013-11-09

**Authors:** Inmaculada Yruela, Bruno Contreras-Moreira

**Affiliations:** 1 Estación Experimental de Aula Dei, Consejo Superior de Investigaciones Científicas (EEAD-CSIC), Avda. Montañana, 1005, Zaragoza 50059, Spain; 2 Institute of Biocomputation and Physics of Complex Systems (BIFI), Universidad de Zaragoza, Mariano Esquillor, Edificio I+D, Zaragoza 50018, Spain; 3 Fundación ARAID, Zaragoza Spain

**Keywords:** Chromosome, Evolution, Intrinsically disordered proteins, Orthologues, Plant genome, Recombination rate

## Abstract

**Background:**

Intrinsically disordered proteins, found in all living organisms, are essential for basic cellular functions and complement the function of ordered proteins. It has been shown that protein disorder is linked to the G + C content of the genome. Furthermore, recent investigations have suggested that the evolutionary dynamics of the plant nucleus adds disordered segments to open reading frames alike, and these segments are not necessarily conserved among orthologous genes.

**Results:**

In the present work the distribution of intrinsically disordered proteins along the chromosomes of several representative plants was analyzed. The reported results support a non-random distribution of disordered proteins along the chromosomes of *Arabidopsis thaliana* and *Oryza sativa*, two model eudicot and monocot plant species, respectively. In fact, for most chromosomes positive correlations between the frequency of disordered segments of 30+ amino acids and both recombination rates and G + C content were observed.

**Conclusions:**

These analyses demonstrate that the presence of disordered segments among plant proteins is associated with the rates of genetic recombination of their encoding genes. Altogether, these findings suggest that high recombination rates, as well as chromosomal rearrangements, could induce disordered segments in proteins during evolution.

## Background

A significant fraction of known eukaryotic genomes encode for proteins that contain regions that do not fold into a well-defined three-dimensional (3D) structure. These proteins are named intrinsically unstructured or disordered proteins (IDPs) and normally carry out signalling and regulatory functions [1-5]. These proteins might be either entirely disordered or partially disordered, characterised by regions spanning just a few (<10) consecutive disordered residues (loops in otherwise well-structured proteins) or long stretches (≥30) of contiguously disordered residues. It is thought that disordered regions confer dynamic flexibility to proteins, allowing transitions between different structural states [6]. The possible utility of such regions was first proposed by Linus Pauling [7]. More recently, computational predictions of disordered regions have discovered that IDPs are prevalent in proteomes, and have increased during evolution. Indeed, it is predicted that 30% to 60% of proteins contain stretches of 30 or more disordered residues, being multicellular eukaryotes more enriched in predicted disordered segments than unicellular eukaryotes and prokaryotes [8]. These results suggest that proteome size, organism complexity and proteome disorder are related. Nevertheless, no overall correlations have been found apart from the clear gain in predicted disorder from prokaryotes to eukaryotes [9]. The relationship between low complexity proteins (LCPs) [10] and recombination rate has been discussed [11]. These authors suggested that the evolution of LCPs in malaria parasite *Plasmodium falciparum* might be related to their high genomic A + T content and recombination rates. However, although low complexity and intrinsic disorder share some structural and sequence similarities, they are distinct phenomena [12]. There is evidence that the unstructured state, common to all living organisms, is essential for basic cellular functions linked with complex responses to environmental stimuli and communication between cells [9-14]. Moreover, structural disorder is critical for some protein-protein interactions, the assembly of large protein complexes and the modulation of protein activity.

The frequency of IDPs in 12 complete plant proteomes, including vascular plants, bryophyte and chlorophyta, has been previously estimated by applying the DISOPRED2 algorithm [15]. That work focused on proteins encoded by genes transferred from the chloroplast to the nucleus and reported a strong correlation between the frequency of disorder of transferred and nuclear-encoded proteins, even for polypeptides that play functional roles back in the chloroplast. Moreover, it suggested that the distribution of disordered and non-disordered segments in proteins could be to some extent random, as it showed that orthologous proteins across different species do not necessarily conserve disordered segments, despite presumably carrying out similar functions.

The evolutionary history of IDPs seems to be multi-parametric, as high disorder content in proteomes has been linked to a variety of observations: *i)* high G + C content, (*i.e.*, in order to explain some exceptions in bacteria, such as *Mycobacterium tuberculosis*, *Myxococcus xanthus* and *Streptomyces coelicolor*) [9,16,17]; *ii)* expanding multi-domain protein families [9]; *iii)* domain arrangements [18]; or *iv)* alternative splicing events [19]. In order to gain additional biological and evolutionary insights into this topic, the frequency of long disordered segments among homologous proteins of 5 plant proteomes is further studied in this work. Likewise, the distribution of IDPs along the chromosomes of model eudicot (*Arabidopsis thaliana*) and monocot (O*ryza sativa*) plant species is analyzed. Finally, the possible correlations between structural disorder, genetic recombination rates and G + C content are investigated.

## Results

### Distribution of disordered segments along proteins

In order to characterize the occurrence of long stretches (L ≥ 30) of contiguously disordered residues among protein domains, their distribution in the proteomes of *A. thaliana* and *O. sativa* was investigated. Obviously, this analysis could only be performed with sequences with both annotated structural domains and predicted disordered segments, which represent 15%-25% of the corresponding proteomes (see Methods). The obtained results indicate that disordered segments generally (*ca.* 90-95%) fall outside protein domains (Table 1). Most of these are actually in the *N*-terminal (42-54%) and *C*-terminal (41-56%) regions, with only (3-7%) of disordered segments sitting in linkers that connect domains.

**Table 1:** **Percentages of disordered residues**^**1 **^**within and outside of protein domains, including linkers, N-terminal and C-terminal regions**

**Chromosomes**	**Proteins**^**2**^	**Total disorder**	**In domains**	**Outside domains**	**linkers**^**3**^	**N-term**^**3**^	**C-term**^**3**^
*Arabidopsis thaliana*							
Chr1	1373	22.29%	6.25%	93.75%	5.97%	48.62%	45.44%
Chr2	770	21.55%	6.46%	93.54%	3.92%	54.20%	41.87%
Chr3	1023	22.22%	5.81%	94.19%	5.16%	47.11%	47.85%
Chr4	816	20.75%	6.78%	93.22%	5.81%	50.38%	43.82%
Chr5	1288	21.11%	4.73%	95.27%	4.74%	42.57%	47.94%
*Oryza sativa*							
Chr1	792	21.36%	3.43%	96,57%	3.43%	47.33%	49.24%
Chr2	672	22.29%	7.21%	92.79%	3.76%	47.09%	49.15%
Chr3	726	21.21%	6.31%	93.69%	4.38%	50.07%	45.61%
Chr4	494	18.35%	6.82%	93.18%	5.92%	49.05%	45.33%
Chr5	465	22.13%	5.84%	94.16%	4.95%	45.85%	49.43%
Chr6	452	22.38%	3.89%	96.11%	7.40%	46.35%	46.68%
Chr7	466	18.97%	5.25%	94.75%	6.48%	45.49%	48.04%
Chr8	365	21.20%	5.37%	94.63%	4.22%	39.97%	55.80%
Chr9	309	20.68%	4.36%	95.64%	4.75%	41.14%	54.19%
Chr10	263	19.67%	8.00%	92.00%	3.08%	47.68%	49.59%
Chr11	243	21.31%	4.17%	95.83%	4.54%	46.22%	49.43%
Chr12	292	21.86%	4.74%	95.26%	1.62%	52.56%	45.85%

^1^Included in segments of ≥ 30 consecutive disordered amino acids over the length of the protein.

^2^Proteins containing Superfamily-defined domains predicted to harbour intrinsically disordered segments.

^3^Calculated on disordered residues outside domains.

### Analysis of disorder in paralogous proteins of plants

The occurrence of protein disorder in paralogous proteins from 5 complete plant proteomes was analyzed (see Methods), including 3 eudicots and 2 monocots species. The total numbers of paralogous protein pairs analyzed were: 4566 from *Arabidopsis thaliana*, 7021 from *Arabidopsis lyrata,* 18641 from *Populus trichocarpa*, 4860 from *Oryza sativa*, and 3096 from *Sorghum bicolor*. The data show that on average 64% of paralogues conserve the number of predicted disordered segments, while 36% gain or lose disordered segments. No differences were observed among the plant proteomes analyzed (Table 2). When *A. thaliana* proteins were annotated with Gene Ontology (GO) terms, paralogous proteins with non-conserved disordered segments were associated with 18 biological processes and 10 molecular functions with corrected *p*-values < 10E-5. As to the biological processes, these paralogues were mainly associated with “regulation”, including regulation of nitrogen compounds (2.64E-20), nucleotide and nucleic acids (1.52E-19), RNA (3.29E-18), macromolecule biosynthesis (4.23E-17) or regulation of gene expression (6.69E-15). The most significant association among specific molecular functions was with “DNA-binding transcription factor activity” (1.71E-25), known to be frequent among IDPs [3,5,14,20].

**Table 2:** Percentages of predicted disordered segments and their conservation across plant paralogues

**Plant proteome**	**Paralogues**	**Conserved disorder**	**Non-conserved disorder**
*A. thaliana*	4566	67%	33%
*A. lyrata*	7021	65%	35%
*P. trichocarpa*	18641	67%	33%
*O. sativa*	4860	62%	38%
*S. bicolor*	3096	60%	40%

**Figure 1:**
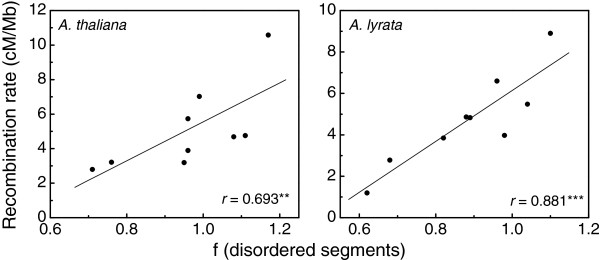
**Scatter plot of frequency of disordered segments in proteins encoded in translocated genomic regions of *A. thaliana *and *A. lyrata *(X-axis) *versus *recombination rates of the corresponding chromosomic regions (Y-axis).** A protein segment is considered disordered if it contains a contiguous stretch of predicted disordered residues of L ≥ 30 amino acids. Statistical significance of Pearson correlation is indicated with *** and **, which correspond to *p* < 0.005 and *p* < 0.01, respectively.

To investigate in more detail these observations a comparative study of *A. thaliana* chromosomes 1 and 4, which correspond to *A. lyrata* chromosomes 1 and 2, and 6 and 7 [21,22], was carried out. Particularly, homologous regions where genome rearrangements (translocations and/or inversions) presumably occurred during evolution were compared. The findings indicate that the percentage of non-conserved disordered proteins which were translocated close to the centromere is lower than that calculated for both *Arabidopsis* complete proteomes (33-35%, Table 2). For instance, the results showed that 2/12 (17%) protein coding genes sitting in the translocated region near the centromere of *A. thaliana* chromosome 4 do not conserve disordered segments. Furthermore, 2/15 (13%) proteins encoded in the S-locus do not conserve the disorder feature (Additional file 1: Figure S1). It can be reasoned that, if both the centromere and the S-locus are reported to have low recombination rates [21,22], these observations could be hinting that the frequency of disorder in proteins might be dependent on the recombination rates of their coding genes. Of course it is important to note that these regions naturally contain much reduced numbers of paralogues, therefore the estimated percentages might be inaccurate. Nevertheless, this hypothesis was tested by plotting the frequency of disordered segments of these *A. thaliana* and *A. lyrata* orthologous proteins *versus* the recombination rates of the corresponding chromosome fragments (Figure 1 and Additional file 1: Table S1). The obtained Pearson correlation coefficients were *r* = 0.693 (*p* < 0.01) and *r* = 0.881 (*p* < 0.005), respectively, indicating that approximately half of the disorder distribution variance can be explained in terms of recombination rates. The comparison could not be extended to other chromosomes due to the lack of reported recombination rates.

**Figure 2:**
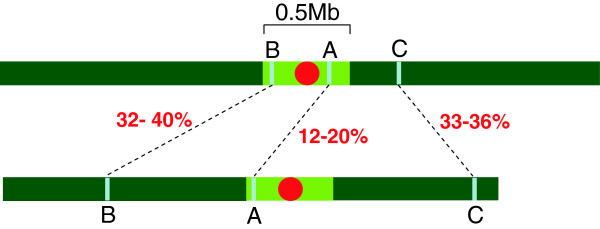
**Diagram of two plant chromosomes with 3 types of paralogues (A, B and C) which differ in their chromosomal location.** Paralogues **A**, **B**, and **C** are marked with blue bars. The percentages of non-conserved disorder between paralogues are shown. The centromere is drawn in red within a centromeric region of 0.5 Mb, shown in light green. The percentage values correspond to 1/8 and 2/10 A-type proteins, 189/463 and 161/505 B-type proteins, and 1419/4056 and 1479/4362 C-type proteins of *A. thaliana* and *O. sativa*, respectively.

Furthermore, in *A. thaliana* and *O. sativa* the results showed that disordered segments are more conserved between paralogues located in regions close to the centromere than those spanning chromosome arms (Figure 2). In particular, disordered segments were not conserved in 1/8 and 2/10 (*ca.* 12-20%) proteins located close to the centromere of *A. thaliana* and *O. sativa*, respectively (A-type proteins in Figure 2). On the contrary, larger values such as 189/463 and 161/505 (*ca*. 32-40%) or 1419/4056 and 1479/4362 (*ca*. 33-36%) were obtained for proteins located in chromosome arms (B-type and C-type proteins*,* re*s*pectively, in Figure 2).

### Genetic recombination correlates with the evolutionary dynamics of disordered segments in A. thaliana and O. sativa

**Figure 3:**
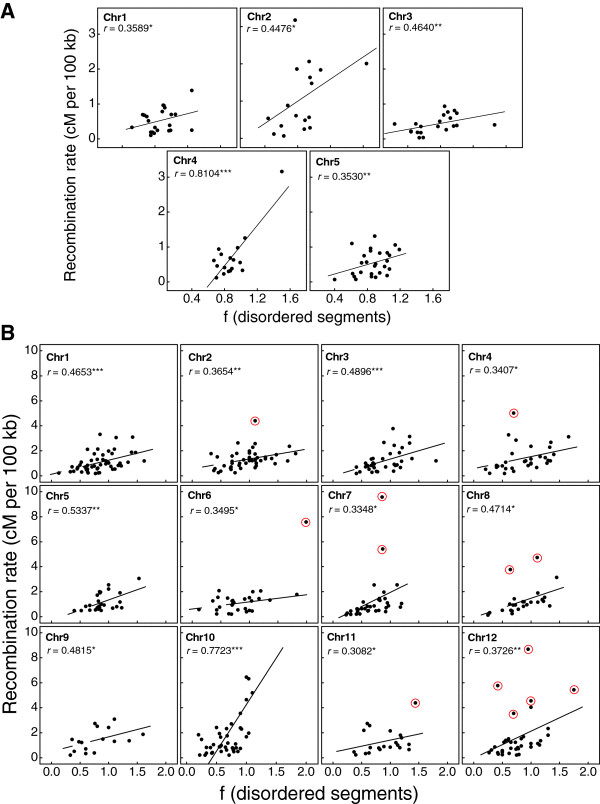
**Scatter plot of frequency of disordered segments in the encoded proteins of mapped regions for each chromosome of *A. thaliana *(A) and *O. sativa *(B) (X-axis) and the corresponding empirical recombination rates (Y-axis).** Proteins with a number of disordered segments higher than the average and associated with hotspots are marked with a red circle. Hotspots in *Oryza sativa* were retrieved from http://rapdb.dna.affrc.go.jp/. Statistical significance of Pearson correlation is indicated with ***, ** and *, which correspond to *p* < 0.005, p < 0.01 and *p* < 0.05, respectively.

The distribution of intrinsic protein disorder along the chromosomes of *A. thaliana* (5) and *O. sativa* (12) was first analyzed by calculating indices of dispersion. Variance-to-mean ratios > 1 were obtained for all chromosomes, suggesting a non-random physical distribution of disordered proteins across the genome. Indeed, a structured distribution could be anticipated as protein disorder has been correlated with high genomic G + C content in other organisms [9]. These results led us to extend the previous analyses of genomic regions within *A. thaliana* and *A. lyrata* to complete plant genomes. For that, the empirical recombination rates reported for 5 chromosomes of *A. thaliana* and 12 chromosomes of *O. sativa* (see Methods) were scatter-plotted *versus* the frequency of disordered segments calculated on those regions (Figure 3A and 3B). The average Pearson correlation coefficients calculated were *r* = 0.487 for *A. thaliana* and *r* = 0.441 for *O. sativa*. These statistically significant correlations show that about a quarter (19-24%) of the overall, genome-scale variance of intrinsic protein disorder distribution can be justified in terms of genetic recombination rates. As expected, this percentage is lower than that calculated for specific homologous regions of *A. thaliana* and *A. lyrata* (see above). However*,* it is noteworthy that a couple of chromosomes (chr 4 of *A. thaliana* and chr 10 of *O. sativa)* showed stronger correlations (*r* = 0.810; *p* < 0.005 and *r* = 0.772; *p* < 0.005), respectively). Outliers, regions where encoded proteins have a larger number of disordered segments (*i.e.* 5–7 segments) than their genomic contexts (*i.e.*, 2–3 segments), were detected. At least in rice, these seem to correspond with recombination hotspots (http://rapdb.dna.affrc.go.jp/).

### G + C content correlates with disordered protein segments in A. thaliana and O. sativa

An investigation about the relationship between gene G + C content and the frequency of disordered residues within the complete proteomes of *A. thaliana* and *O. sativa* was carried out. The G + C content of complete nucleotide sequences (G + C_total_), disordered (G + C_disordered_) and ordered (G + C_ordered_) segments was calculated for all predicted IDPs (Additional file 1: Table S2). The results show that disordered regions are modestly but significantly enriched in G + C bases (+3.0% in *O. sativa* and +1.6% in *A. thaliana*, *p* < 0.01). Similar enrichments were observed in *Sorghum bicolor* (monocot) and *Arabidopsis lyrata* and *Populus trichocarpa* (eudicots) (Additional file 1: Table S2).

The G + C_disordered_ frequency was plotted *versus* the frequency of disordered residues calculated for chromosome windows in monocot (2) and eudicot (3) plant species (Additional file 1: Figure S2). In the case of *A. thaliana* the obtained Pearson correlation coefficients were between *r* = 0.773 (*p* < 1E-4) for chromosome 5 and *r* = 0.928 (*p* < 1E-4) for chromosome 3. In the case of *O. sativa* the correlation coefficients were between *r* = 0.737 (*p* < 1E-4) for chromosome 8 and *r* = 0.869 (*p* < 1E-4) for chromosome 11. These results unveil a strong dependence (*r*^2^ = 0.78 for *A. thaliana* and *r*^2^ = 0.66 for *O. sativa*) of these two variables. Similar results were obtained for the eudicots *A. lyrata* (*r*^2^ = 0.84) and *P. trichocarpa* (*r*^2^ = 0.76) and the monocot *S. bicolor* (*r*^2^ = 0.80).

### G + C content correlates with recombination rates in A. thaliana and O. sativa

**Figure 4:**
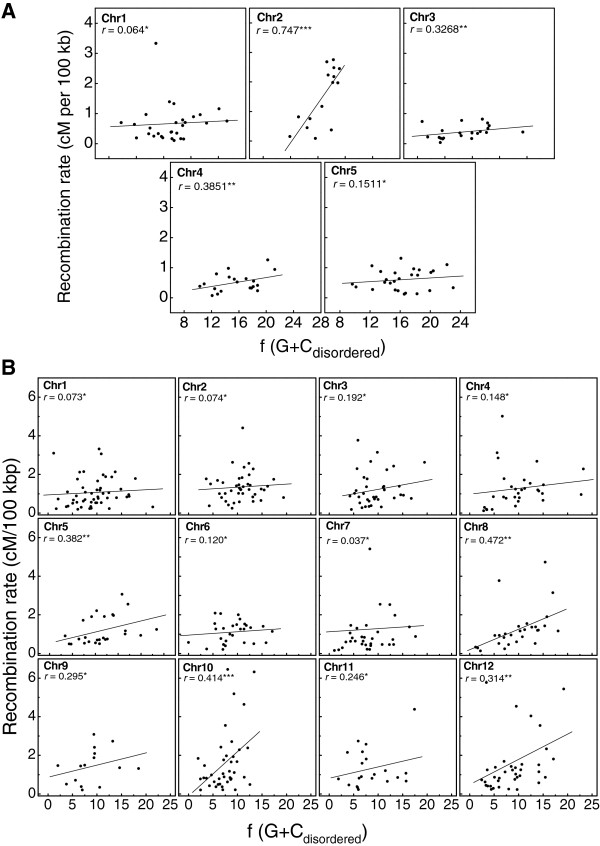
**Scatter plots of G** + **C **_**disordered **_**frequencies in genes of mapped regions for each chromosome of *A. thaliana *(A) and *O. sativa *(B) (X-axis) *versus *the corresponding empirical recombination rates (Y-axis).** Statistical significance of Pearson correlation is indicated with ***, ** and *, which correspond to *p* < 0.005, *p* < 0.01 and *p* < 0.05, respectively.

The fact that the occurrence of protein disorder is to some extent related to recombination rates and G + C content could suggest that these two later variables might also be linked. The tests carried out in this work show that they are significantly but weakly correlated, with average coefficients of *r* = 0.334 in *A. thaliana* and *r* = 0.231 in *O. sativa* (Figure 4A and 4B).

In order to re-assess the dependencies among protein disorder, G + C content and recombination rates, a multiple regression analysis was performed. Taking the *A. thaliana* data a linear model was obtained with multiple *r*^2^ = 0.52, indicating that protein disorder was only significantly dependent on G + C content (*p* < 2E-16). The rice model (*r*^2^ = 0.46) confirmed the main contribution of G + C content to protein disorder (*p* < 2E-16), but also supported a minor, but significant role of recombination rates (*p* = 0.013). Note that gene density within *A. thaliana* chromosomal regions where recombination rates have been reported is about an order of magnitude larger than in *O. sativa* (see Methods). These analyses required the calculation of frequencies of disordered residues as explained in Methods (Additional file 1: Figures S3A and S3B).

## Discussion

In a previous paper, the analysis of 12 plant proteomes revealed a similar occurrence of IDPs to that found in other eukaryotic organisms [15], and concerning their taxonomic distribution, no differences were observed for IDPs among plant species. However, in some cases, homologous sequences displayed variations in the frequency of disordered segments. The inspection of 5 representative plant proteomes performed in this work indicated that on average 36% of paralogues do not conserve their composition of disordered segments. These proteins seem to be involved in regulatory processes, as most IDPs are, and therefore there is no obvious functional argument to explain their differential conservation behaviour. This result fits well with a previous study in yeast, which reported that non-conserved disordered proteins cannot be clearly associated with any function, and are expressed at low levels [5].

Gene duplication is a prominent feature in plant genome evolution with likely implications in genetic diversity and adaptation, although there is not a direct causal link between an adaptive phenotype and a specific gene duplication event because they usually occur at different times [23]. Duplicate genes arise either by regional genomic events or genome-wide polyploidization. In plants, the last is the most common mechanism. For instance, in *Arabidopsis,* duplications most probably resulted from a single tetraploidization event occurred some 65 million years ago [24]. This phenomenon presumably involved most genomic regions, although it has been found that centromeric regions have significantly fewer duplicated genes than chromosome arms [25,26]. In addition to these events, which are charted in physical maps, the available genetic maps expose the empirical recombination rates along each chromosome. It is known that recombination rates vary substantially along genomic regions. For instance, the average recombination rate ranges from 0.3 cM/Mb to 251 cM/Mb in *A. thaliana*[17] and from 0.39 to 0.42 cM/Mb in *O. sativa*[27]. Peak recombination rates can indicate hotspots, which are opposed to regions of suppressed recombination (coldspots). An overall positive correlation between gene density and recombination rate has been reported in model plant *Brachypodium distachyon*. On the contrary, a negative correlation has been observed between gene density and the frequency of repetitive regions, and rearranged chromosomal segments that retained centromeric repetitive sequences [28].

The analyses reported in this work show, for the first time, positive correlations between genetic recombination rates and protein disorder frequency in *A. thaliana* and *O. sativa*. Moreover, the results expose that certain proteins with substantially more predicted disordered segments (*i.e.,* 5*–*7 segments) than the average (*i.e.,* 2*–*3 segments) are located within recombination hotspots [29] (Figure 3B). These findings suggest that the physical location of paralogous genes along chromosomes could partially explain the differences found in their protein disorder composition. Genetic recombination could then be considered an evolutionary force contributing to structural disorder in proteins, at least in plants. Previous reports already discussed a relationship between low complexity proteins (LCPs) and recombination rate in *Plasmodium falciparum*[11]. Interestingly, in this parasite up to 50% proteins are longer than their yeast orthologues due likely to insertions or expansions of LC regions [30].

Changes in genomic architecture are a formidable force in the evolution of plants, and structural chromosome rearrangements similar to those of *A. lyrata* and *A. thaliana*[21,22] are frequent. As a side-effect, these processes can drive domain sorting in proteins or the formation of novel domains [31]. Indeed, it has been reported that a significant portion of emerged novel domains during evolution are highly disordered [18]. Thus, evolutionary increase of protein disorder could be driven by modular or domain exchanges. The link between intrinsic structural disorder and modularity has been recently investigated in the human genome, finding that high levels of disorder within proteins are encoded by symmetric exons, possibly derived from internal tandem duplications [32]. The data in this work clearly indicate that disordered segments are mostly located outside annotated domains, with a similar frequency at both *N*- and *C*- termini, and a rather low occurrence in linker regions.

This paper reports strong positive correlations between G + C content in coding sequences and predicted protein disorder in 5 plant proteomes. This finding is in agreement with computational studies in Archaea and Bacteria, which established relationships between G + C composition and intrinsic protein disorder [33]. During meiotic recombination, parental chromosomes undergo either large-scale genetic exchanges by crossover or small-scale exchanges by gene conversion. There is evidence that in some eukaryote species gene conversion affecting G/C:A/T heterozygous sites yields more frequently G/C than A/T alleles. This process is known as GC-biased gene conversion (gBGC) and increases the GC content of recombining DNA over evolutionary time [34-37]. Indeed gBGC is considered the major mechanism explaining the variation of G + C content within and between eukaryote genomes, as coding sequences rich in G + C bases have a higher content of Arg, Gly, Ala and Pro codons, precisely those amino acids overrepresented in IDPs [2,15,33,38]. These composition differences explain the G + C content reported in this work for ordered and disordered regions.

Previous papers have published strong positive correlations in human, yeast, *Caenorhabditis elegans*, *Drosophila melanogaster* and two rice species between crossover rates and G + C composition [39-42]. On the contrary, the work of Wu *et al.*[27] about recombination hotspots and coldspots in *O. sativa* did not reveal a clear relationship between these two variables. Moreover, Pessia *et al*. [43] found no significant correlations in the genomes of *A. thaliana, P. trichocarpa* and *Vitis vinifera* and even reported a negative correlation not consistent with gBGC in *S. bicolor*. A negative correlation was also reported for *A. thaliana* chromosome 4 [44]. At first sight these apparent contradictions could be telling that the relationship between recombination and G + C composition might be dependent on the plant species. Yet, a review of these studies reveals that G + C measurements are not always comparable, and that recombination rates are estimated with different resolution thresholds. For instance, theoretical equilibrium G + C values cannot be directly compared to empirical G + C counts in sequenced genomes. Regarding this open question, this paper reports a significant but weak association between recombination and G + C content in *A. thaliana* and *O. sativa*. When a multiple regression analysis was carried out to delineate their influence on protein disorder, clearly the effect of G + C content was stronger than recombination. Taken together, these observations support a strong molecular-based dependency of protein disorder and G + C content, while suggesting a much weaker relationship between G + C and recombination. In other words, codon composition of amino acid residues common in disordered segments is directly translated into higher G + C values. However, the proposed link between gBGC and G + C content is much harder to capture with the kind of data used in this work.

## Conclusion

The results demonstrate that the presence of disordered segments among plant protiens is associated with the rates of genetic recombination of their encoding genes. High recombination rates, as well as chromosomal rearrangements, could induce disordered segments in proteins during evolution. Additionally, the results indicated a stronger molecular-based dependency of protein disorder and G + C content and much weaker dependency between G + C content and recombination rate in plant genomes.

## Methods

### Proteomic, GO and chromosome map databases

Complete plant proteomes, orthology and paralogy assignments and GO annotations for *Arabidopsis thaliana* (AT), *Oryza sativa* (OS)*, Populus trichocarpa* (PT)*, Sorghum bicolor* (SB) were retrieved from PLAZA v.1 (http://bioinformatics.psb.ugent.be/plaza_v1/), and *Arabidopsis lyrata* (AL) from PLAZA v.2.5 (http://bioinformatics.psb.ugent.be/plaza/). Note that the same data versions of a previous paper [15] were employed to facilitate comparisons to the results published here. Genetic maps from *A. thaliana* and *O. sativa* were retrieved from http://www.arabidopsis.org/servlets/mapper and http://rapdb.dna.affrc.go.jp/, respectively. Superfamily-defined (http://supfam.cs.bris.ac.uk) protein domains for *A. thaliana* and *O. sativa* were retrieved from http://bioinformatics.psb.ugent.be.

### Recombination rates

Empirical rates of recombination in *A. thaliana* and *O. sativa* were taken from Colomé-Tatché *et al.*[29] and IRGSP/RAP build 5 annotation data (http://rapdb.dna.affrc.go.jp/), respectively. For Pearson correlation analyses, chromosomes were split in fragments corresponding to the regions of the genetic map where recombination rates had been empirically determined. The mean sizes of those fragments were 0.83 kb ± 0.14 and 0.14 ± 0.005 kb for *A. thaliana* and *O. sativa*, respectively. The mean numbers of contained genes were 178 ± 36 and 19 ± 7, respectively. Recombination rates (cM) were normalized by dividing by window size (Mb).

### Predictions of intrinsic disorder

DISOPRED2 v2.42 [45] disorder predictions were performed for all protein sequences annotated in 5 plant species. All input sequences, plus the reference database *uniref90*, were low-complexity filtered with PFILT and scanned with 3 iterations of *blastpgp* with an E-value cut-off of 0.001. Please check the previous paper for a benchmark on disorder predictions in plant proteins [15]. In order to put these results on a genomic scale, and to correct for regions with distinct gene density, chromosomes were split in non-overlapping windows and the observed number of disordered segments of length (L) ≥30 amino acids in each window divided by the total number of open reading frames contained therein.

Frequencies of disordered residues were also computed for their direct comparison with windowed G + C contents, which are residue-based, in a multiple regression analysis, as explained below. These frequencies were defined as the number of disordered residues contained in segments of L ≥ 30 over the length of the encoding gene. It is worth mentioning that linear regressions using frequencies of disordered residues yield coefficients somewhat lower than those shown in Figure 3.

### Calculation of G + C content

Three variants of G + C content were computed: i) G + C_total_, the total number of G + C bases over the complete nucleotide sequence of a gene; ii) G + C_disordered_, the number of G + C bases spanning the predicted disordered segments in a gene; and iii) G + C_ordered_ = G + C_total_ - G + C_disordered_. The G + C_disordered_ frequency was defined as G + C_disordered_ / gene_length.

### Statistical analyses

Student’s *t* tests on G + C content were performed after checking the normality of data. The index of dispersion is defined as the ratio of the variance to the mean physical location of annotated genes, and was calculated for individual chromosomes. Multiple regression linear models of frequency of disordered residues as a function of both G + C content and recombination rates were calculated with the *lm* function of the R package (http://www.R-project.org) with *A. thaliana* and *O. sativa* data. The obtained linear models were subsequentially evaluated with ANOVA tests in order to assess the contribution of each variable. Heteroscedasticity and normality diagnostic plots were performed to validate the model.

## Competing interests

The authors declare that they have no competing interests.

## Authors’ contributions

IY carried out the genome analysis, participated in the design and coordination of the study, and wrote the manuscript. BC-M participated in the design of the study and the data analysis, and helped write the manuscript. Both authors have read and approved the final manuscript.

## Supplementary Material

Additional file 1: Figure S1 Diagram of *A. thaliana* chromosome 4 and the corresponding regions of *A. lyrata* chromosomes 6 and 7. The number of proteins in S-locus and translocated regions near the centromere, and the corresponding percentages of non-conserved disorder, are shown. Accession codes of proteins in S-locus region: At4g19680 (*), At4g20360, At4g20410, At4g20760, At4g20960, At4g21150 (*), At4g21340, At4g21350, At4g21430, At4g21580, At4g21800, At4g21960, At4g22200, At4g22360, At4g22720. Accession codes of proteins in the translocated region: At4g00030, At4g00660 (*), At4g02390, At4g04350, At4g05420, At4g12030, At4g10340, At4g08170 (*), At4g07390, At4g06744, At4g06599, At4g06534. Proteins marked with (*) do not conserve disordered segments. **Table S1.** Recombination rates and frequency of disordered segments in *A. thaliana* chromosomes 1 and 4, and the corresponding regions of *A. lyrata* chromosomes 1 and 2, and of *A. lyrata* chromosomes 6 and 7. **Table S2.** Percentages of G + C content in the nucleotide sequences of encoded proteins, ordered and disordered regions. **Figure S2.** Scatter plot of disordered residue frequencies in proteins from each chromosome from *A. thaliana* (A), *A. lyrata* (B), *O. sativa* (C), *P. trichocarpa* (D) and *S. bicolor* (E) (X-axis) *versus* the G + C_disordered_ frequency of their gene coding sequence (Y-axis). Disordered residue frequencies were calculated as the number of residues in the disordered segments of length (L) ≥30 amino acids within 0.5 Mb windows divided by the total number of residues in the open reading frames. Statistical significance of Pearson correlation is indicated with ***, ** and *, which correspond to *p* < 0.005, *p* < 0.01 and *p* < 0.05, respectively. **Figure S3.** Scatter plot of disordered residue frequency in the encoded proteins of mapped regions for each chromosome of *A. thaliana* (A) and *O. sativa* (B) (X-axis) *versus* the corresponding empirical recombination rates (Y-axis). Disordered residue frequencies were calculated as the number of residues in disordered segments of length (L) ≥30 amino acids in each mapped chromosomic region divided by the total number of residues in the open reading frames. Statistical significance of Pearson correlation is indicated with ***, ** and *, which correspond to *p* < 0.005, *p* < 0.01 and *p* < 0.05, respectively. Click here for file
