# Supplementary material for: Genetic recombination is associated with intrinsic disorder in plant proteomes
Source: BMC Genomics. 2013 Nov 9;14:772. doi: 10.1186/1471-2164-14-772 (PMC3828576; doi:10.1186/1471-2164-14-772)
Supplement: Additional file 1: Figure S1 — Diagram of A. thaliana chromosome 4 and the corresponding regions of A. lyrata chromosomes 6 and 7. The number of proteins in S-locus and translocated regions near the centromere, and the corresponding percentages of non-conserved disorder, are shown. Accession codes of proteins in S-locus region: At4g19680 (*), At4g20360, At4g20410, At4g20760, At4g20960, At4g21150 (*), At4g21340, At4g21350, At4g21430, At4g21580, At4g21800, At4g21960, At4g22200, At4g22360, At4g22720. Accession codes of proteins in the translocated region: At4g00030, At4g00660 (*), At4g02390, At4g04350, At4g05420, At4g12030, At4g10340, At4g08170 (*), At4g07390, At4g06744, At4g06599, At4g06534. Proteins marked with (*) do not conserve disordered segments. Table S1. Recombination rates and frequency of disordered segments in A. thaliana chromosomes 1 and 4, and the corresponding regions of A. lyrata chromosomes 1 and 2, and of A. lyrata chromosomes 6 and 7. Table S2. Percentages of G + C content in the nucleotide sequences of encoded proteins, ordered and disordered regions. Figure S2. Scatter plot of disordered residue frequencies in proteins from each chromosome from A. thaliana (A), A. lyrata (B), O. sativa (C), P. trichocarpa (D) and S. bicolor (E) (X-axis) versus the G + Cdisordered frequency of their gene coding sequence (Y-axis). Disordered residue frequencies were calculated as the number of residues in the disordered segments of length (L) ≥30 amino acids within 0.5 Mb windows divided by the total number of residues in the open reading frames. Statistical significance of Pearson correlation is indicated with ***, ** and *, which correspond to p < 0.005, p < 0.01 and p < 0.05, respectively. Figure S3. Scatter plot of disordered residue frequency in the encoded proteins of mapped regions for each chromosome of A. thaliana (A) and O. sativa (B) (X-axis) versus the corresponding empirical recombination rates (Y-axis). Disordered residue frequencies were calculated as the number of residues in diso [file 1471-2164-14-772-S1.pdf]

**Additional file 1:**

**Figure S1.-** Diagram of *A. thaliana* chromosome 4 and the corresponding regions of *A. lyrata* chromosomes 6 and 7. The number of proteins in S-locus and translocated regions near the centromere, and the corresponding percentages of non-conserved disorder, are shown. Accession codes of proteins in S-locus region: At4g19680 (*), At4g20360, At4g20410, At4g20760, At4g20960, At4g21150(*), At4g21340, At4g21350, At4g21430, At4g21580, At4g21800, At4g21960, At4g22200, At4g22360, At4g22720. Accession codes of proteins in the translocated region: At4g00030, At4g00660(*), At4g02390, At4g04350, At4g05420, At4g12030, At4g10340, At4g08170(*), At4g07390, At4g06744, At4g06599, At4g06534. Proteins marked with (*) do not conserve disordered segments.

**Table S1.-** Recombination rates and frequency of disordered segments in *A. thaliana* chromosomes 1 and 4, and the corresponding regions of *A. lyrata* chromosomes 1 and 2, and of *A. lyrata* chromosomes 6 and 7.

**Table S2.-** Percentages of G+C content in the nucleotide sequences of encoded proteins, ordered and disordered regions.

**Figure S2.-** Scatter plot of disordered residue frequencies in proteins from each chromosome from *A. thaliana* (A), *A. lyrata* (B), *O. sativa* (C), *P. trichocarpa* (D) and *S. bicolor* (E) (X-axis) *versus* the $G+C_{disordered}$ frequency of their gene coding sequence (Y-axis). Disordered residue frequencies were calculated as the number of residues in the disordered segments of length (L) ≥30 amino acids within 0.5Mb windows divided by the total number of residues in the open reading frames. Statistical significance of Pearson correlation is shown.

**Figure S3.**- Scatter plot of disordered residue frequency in the encoded proteins of mapped regions for each chromosome of *A. thaliana* (A) and *O. sativa* (B) (X-axis) *versus* the corresponding empirical recombination rates (Y-axis). Disordered residue frequencies were calculated as the number of residues in disordered segments of length (L) ≥30 amino acids in each mapped chromosomic region divided by the total number of residues in the open reading frames. Statistical significance of Pearson correlation is indicated with ***, ** and *, which correspond to $p<0.005$, $p<0.01$ and $p<0.05$, respectively.

## Comparison between *A. lyrata* and *A. thaliana*

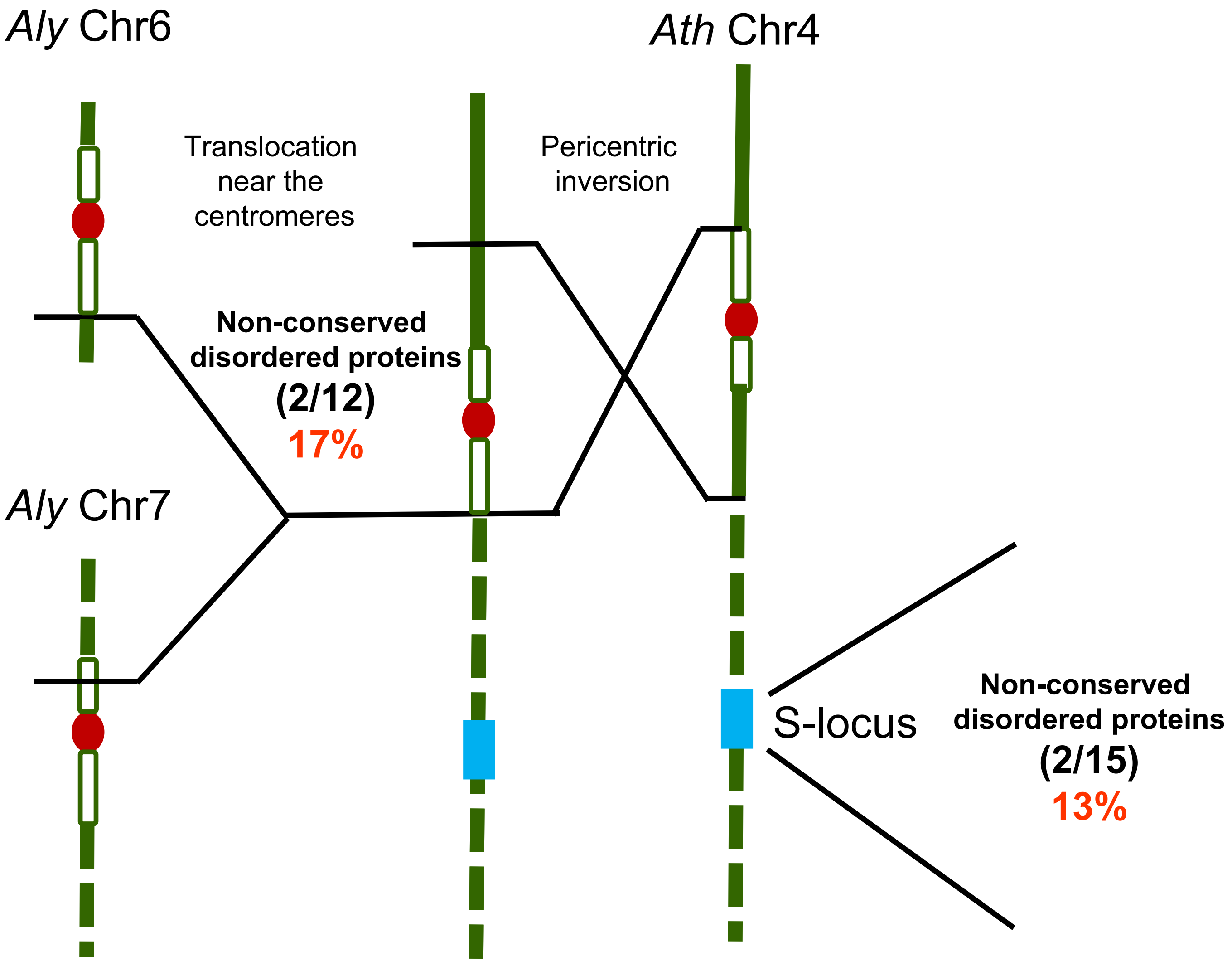


Figure S1

**Table S1.-** Recombination rates and average disorder of *A. thaliana* chromosome 1 and 4, and the corresponding regions of *A. lyrata* chromosomes 1 and 2, and of *A. lyrata* chromosomes 6 and 7.

| | *Arabidopsis thaliana* | | | | *Arabidopsis lyrata* | | | |
|---|---|---|---|---|---|---|---|---|
| Chromosome region [1] | genes | cM/Mb | average disorder[2] | %G+C | genes | cM/Mb | average disorder[2] | %G+C |
| *A. thaliana* Chr 1 | | | | | | | | |
| *A. lyrata* Chr1 | 4582 | 3.89 | 0.96 | 0.443 | 4921 | 3.97 | 0.98 | 0.444 |
| short arm | 3480 | 4.68 | 1.08 | 0.447 | 3662 | 4.83 | 0.89 | 0.447 |
| long arm | 1010 | 4.76 | 1.11 | 0.436 | 1168 | 3.84 | 0.82 | 0.437 |
| | | | | | | | | |
| *A. lyrata* Chr2 | 1381 | 7.02 | 0.99 | 0.442 | 1645 | 8.90 | 0.86 | 0.443 |
| *A. lyrata* Chr2 inverted region | 390 | 3.19 | 0.95 | 0.435 | 519 | 5.48 | 1.04 | 0.576 |
| | | | | | | | | |
| *A. thaliana* Chr 4 | | | | | | | | |
| *A. lyrata* Chr6 derived (non-inverted) | 554 | 10.58 | 1.17 | 0.438 | 430 | 6.60 | 0.96 | 0.448 |
| *A. lyrata* Chr6 derived (non-inverted) | 347 | 2.79 | 0.71 | 0.549 | 375 | 2.78 | 0.68 | 0.636 |
| | | | | | | | | |
| *A. lyrata* Chr 7-derived | 2847 | 5.73 | 0.96 | 0.447 | 2784 | 4.86 | 0.88 | 0.448 |
| S-locus region | 322 | 3.21 | 0.76 | 0.444 | 329 | 1.19 | 0.62 | 0.441 |

[1] Kawabe *et al.* (2006) Genetical Research 88: 45-56.
[2] A protein is considered disordered if it contains a contiguous stretch of predicted disordered residues of L ≥ 30 amino acids.

**TABLE S2.- Percentages of G+C content in the nucleotide sequence of encoded proteins, ordered and disordered regions[1]**

| Chromosomes | n genes | GCtotal/Ntotal | GCord/Nord | GCdiso/Ndiso |
|---|---|---|---|---|
| *Arabidopsis thaliana* | | | | |
| Chr1 | 4210 | 44.07% | 43.80% | 45.41% |
| Chr2 | 7005 | 44.17% | 43.88% | 45.54% |
| Chr3 | 5398 | 44.25% | 43.95% | 45.74% |
| Chr4 | 4104 | 44.19% | 43.89% | 45.72% |
| Chr5 | 6306 | 44.07% | 43.83% | 45.19% |
| *Arabidopsis lyrata* | | | | |
| Chr1 | 5376 | 45.55% | 43.99% | 45.55% |
| Chr2 | 3004 | 43.88% | 43.58% | 45.61% |
| Chr3 | 4214 | 44.41% | 44.12% | 45.94% |
| Chr4 | 3638 | 44.38% | 44.07% | 45.90% |
| Chr5 | 3471 | 44.16% | 43.87% | 45.79% |
| Chr6 | 4177 | 44.47% | 44.24% | 45.65% |
| Chr7 | 4108 | 44.28% | 43.98% | 45.91% |
| Chr8 | 3474 | 44.03% | 43.71% | 45.62% |
| *Oryza sativa* | | | | |
| Chr1 | 5410 | 55.37% | 54.88% | 57.95% |
| Chr2 | 4381 | 54.92% | 54.40% | 57.44% |
| Chr3 | 4609 | 55.63% | 55.24% | 57.39% |
| Chr4 | 3667 | 55.11% | 54.57% | 58.14% |
| Chr5 | 3336 | 57.75% | 55.31% | 57.80% |
| Chr6 | 3492 | 55.93% | 55.54% | 57.95% |
| Chr7 | 3334 | 55.55% | 55.15% | 57.79% |
| Chr8 | 2955 | 55.18% | 54.71% | 57.56% |
| Chr9 | 2448 | 55.45% | 54.94% | 58.08% |
| Chr10 | 2453 | 55.69% | 55.20% | 58.70% |
| Chr11 | 3001 | 53.51% | 52.96% | 57.26% |
| Chr12 | 2667 | 54.43% | 53.96% | 56.93% |

| | | | | |
|---|---|---|---|---|
| *Populus trichocarpa* | | | | |
| Chr1 | 2993 | 43.62% | 43.38% | 44.90% |
| Chr2 | 2553 | 44.09% | 43.86% | 45.20% |
| Chr3 | 1761 | 43.65% | 43.42% | 44.91% |
| Chr4 | 1340 | 43.45% | 43.23% | 44.86% |
| Chr5 | 1603 | 43.90% | 43.62% | 45.25% |
| Chr6 | 1881 | 43.49% | 43.26% | 44.67% |
| Chr7 | 1204 | 43.47% | 43.22% | 44.84% |
| Chr8 | 1881 | 44.10% | 43.86% | 45.32% |
| Chr9 | 1534 | 43.92% | 43.66% | 45.32% |
| Chr10 | 2192 | 43.93% | 43.67% | 45.30% |
| Chr11 | 1175 | 43.23% | 42.95% | 44.99% |
| Chr12 | 1214 | 43.51% | 43.28% | 44.75% |
| Chr13 | 1082 | 43.28% | 43.05% | 44.61% |
| Chr14 | 1345 | 44.06% | 43.83% | 45.31% |
| Chr15 | 1156 | 43.52% | 43.33% | 44.87% |
| Chr16 | 1219 | 43.66% | 43.41% | 45.11% |
| Chr17 | 420 | 43.33% | 43.06% | 44.87% |
| Chr18 | 1152 | 43.57% | 43.29% | 45.28% |
| *Sorghum bicolor* | | | | |
| Chr1 | 5271 | 55.02% | 54.61% | 56.69% |
| Chr2 | 4277 | 55.51% | 54.72% | 57.20% |
| Chr3 | 4348 | 55.09% | 54.64% | 57.30% |
| Chr4 | 3529 | 54.62% | 54.11% | 56.96% |
| Chr5 | 2495 | 53.21% | 52.69% | 56.40% |
| Chr6 | 2860 | 54.44% | 53.97% | 56.90% |
| Chr7 | 2322 | 54.13% | 53.72% | 56.11% |
| Chr8 | 2038 | 53.12% | 52.61% | 55.67% |
| Chr9 | 2576 | 54.75% | 54.27% | 56.81% |
| Chr10 | 2781 | 54.94% | 54.66% | 56.30% |

[*] t-Student statistic indicates that at the 0.05 level the means are significantly different.

[1] Ntotal, nucleotides in encoded proteins; Nord, nucleotides in ordered regions; Ndiso, nucleotides in disordered regions; GCtotal, G+C content in encoded proteins; GCord, G+C content in ordered regions; GCdiso, G+C content in disordered regions;

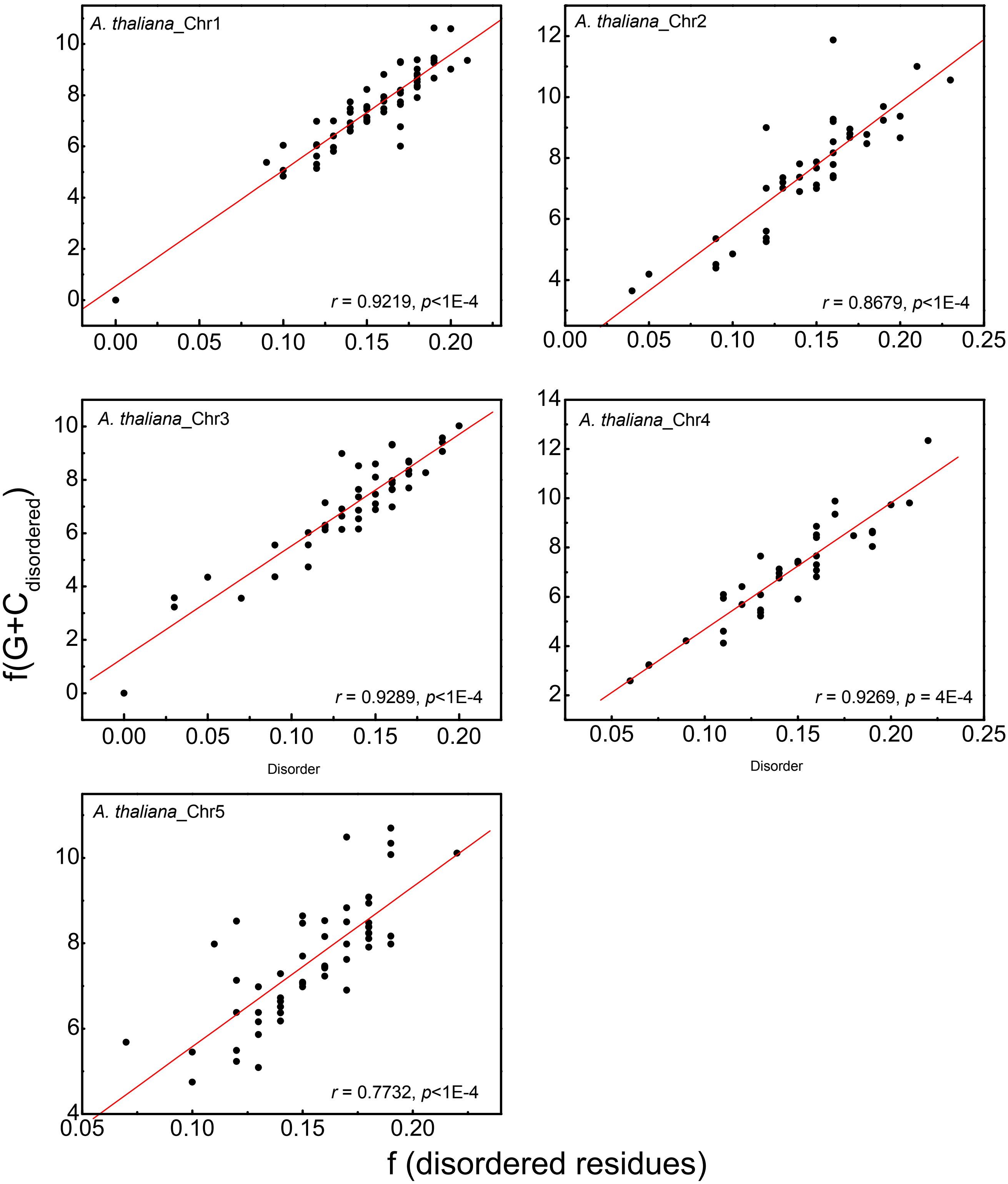

A. thaliana_Chr1
r = 0.9219, p<1E-4
A. thaliana_Chr2
r = 0.8679, p<1E-4
A. thaliana_Chr3
r = 0.9289, p<1E-4
Disorder
A. thaliana_Chr4
r = 0.9269, p = 4E-4
Disorder
A. thaliana_Chr5
r = 0.7732, p<1E-4
f(G+C disordered)
f (disordered residues)


Figure 2A

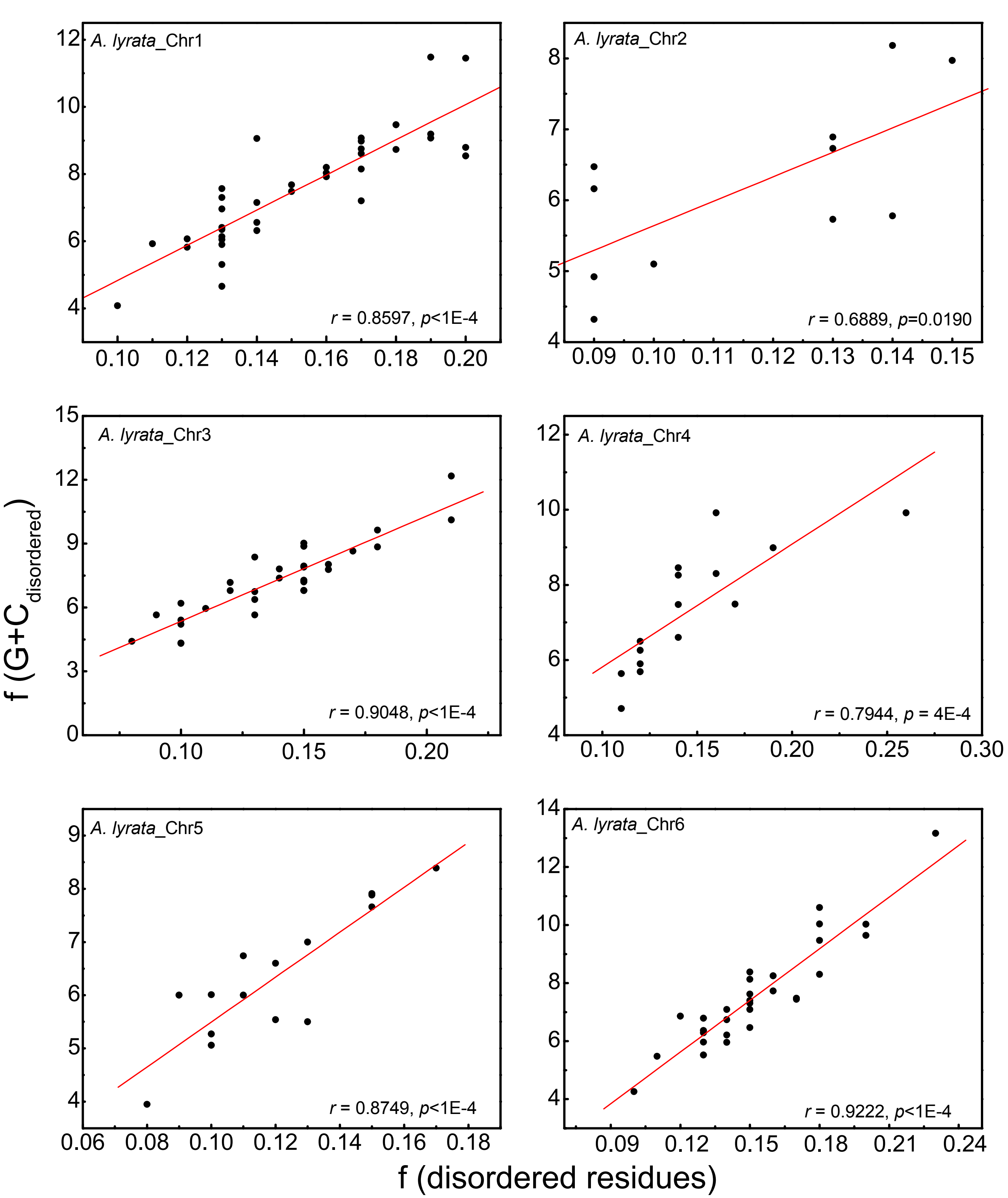
A. lyrata_Chr1
r = 0.8597, p<1E-4
A. lyrata_Chr2
r = 0.6889, p=0.0190
A. lyrata_Chr3
r = 0.9048, p<1E-4
A. lyrata_Chr4
r = 0.7944, p = 4E-4
A. lyrata_Chr5
r = 0.8749, p<1E-4
A. lyrata_Chr6
r = 0.9222, p<1E-4
f (G+C disordered)
f (disordered residues)

Figure 2B

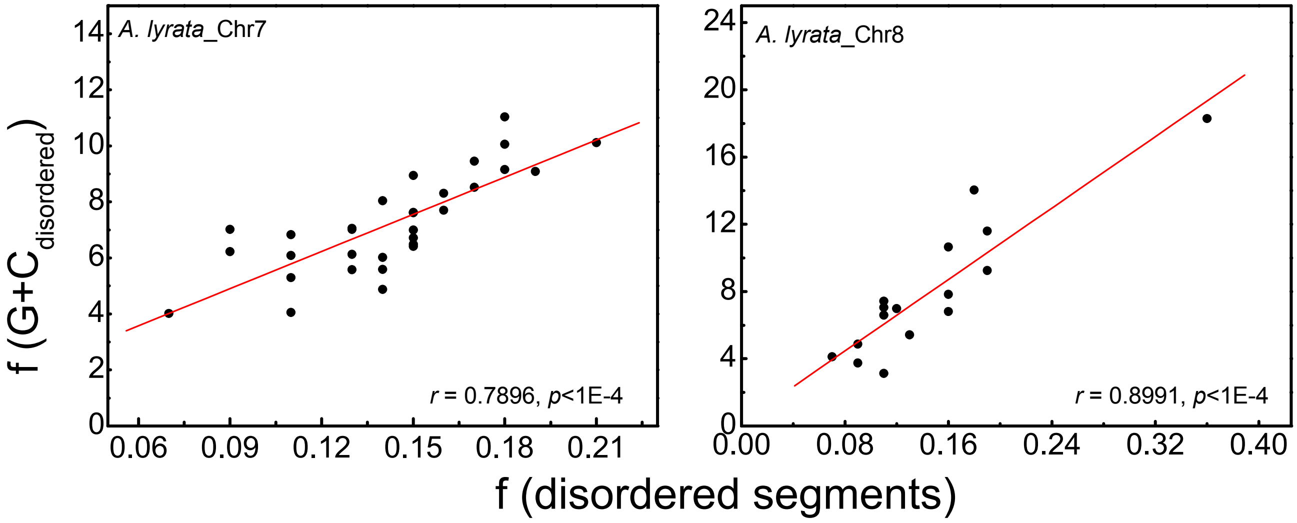
A. lyrata_Chr7
r = 0.7896, p<1E-4
A. lyrata_Chr8
r = 0.8991, p<1E-4
f (G+C$_{disordered}$)
f (disordered segments)
0
2
4
6
8
10
12
14
0.06
0.09
0.12
0.15
0.18
0.21
0
4
8
12
16
20
24
0.00
0.08
0.16
0.24
0.32
0.40

Figure 2B

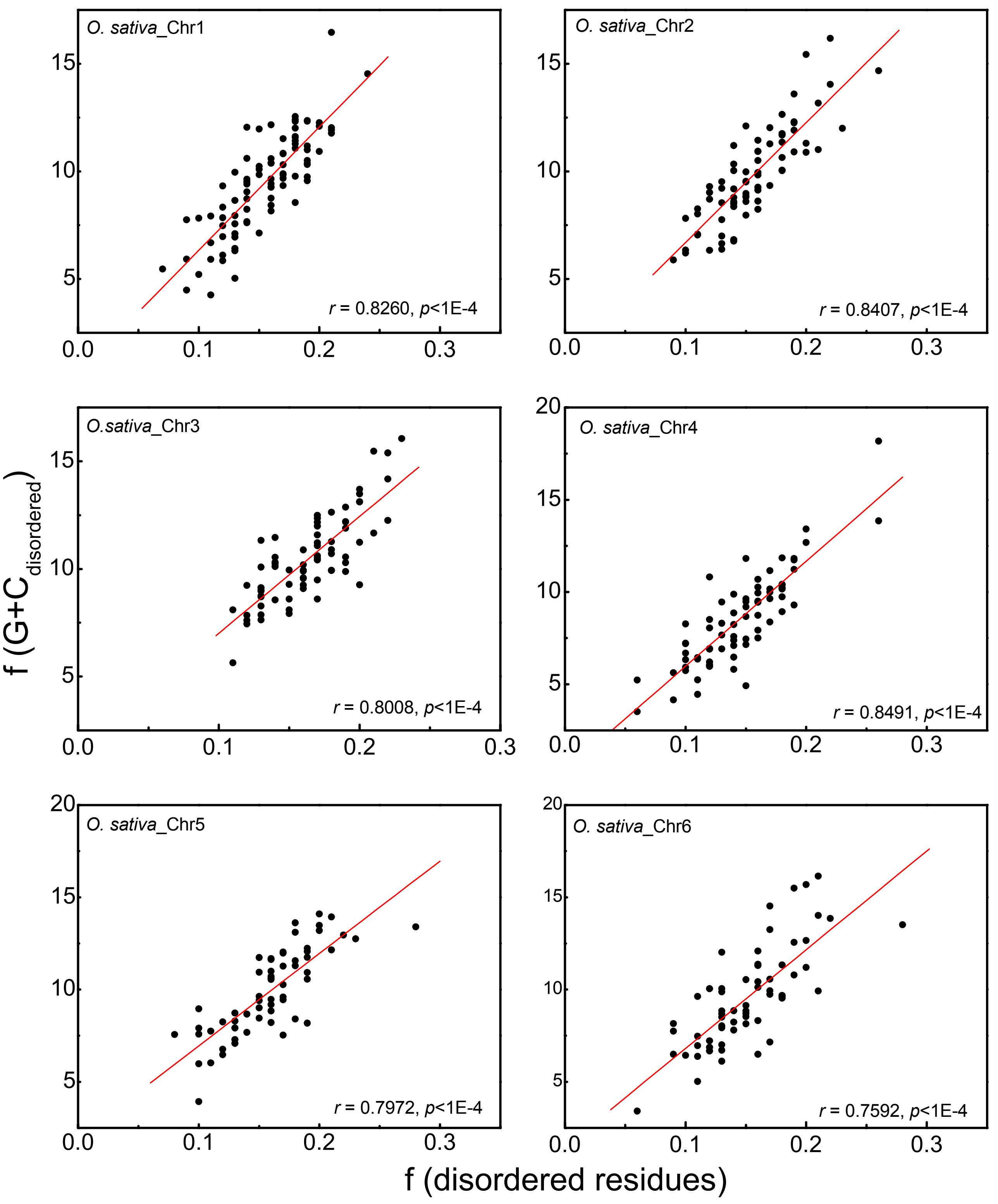
O. sativa_Chr1
r = 0.8260, p<1E-4
O. sativa_Chr2
r = 0.8407, p<1E-4
O.sativa_Chr3
r = 0.8008, p<1E-4
O. sativa_Chr4
r = 0.8491, p<1E-4
O. sativa_Chr5
r = 0.7972, p<1E-4
O. sativa_Chr6
r = 0.7592, p<1E-4
f (G+C disordered)
f (disordered residues)

Figure S2C

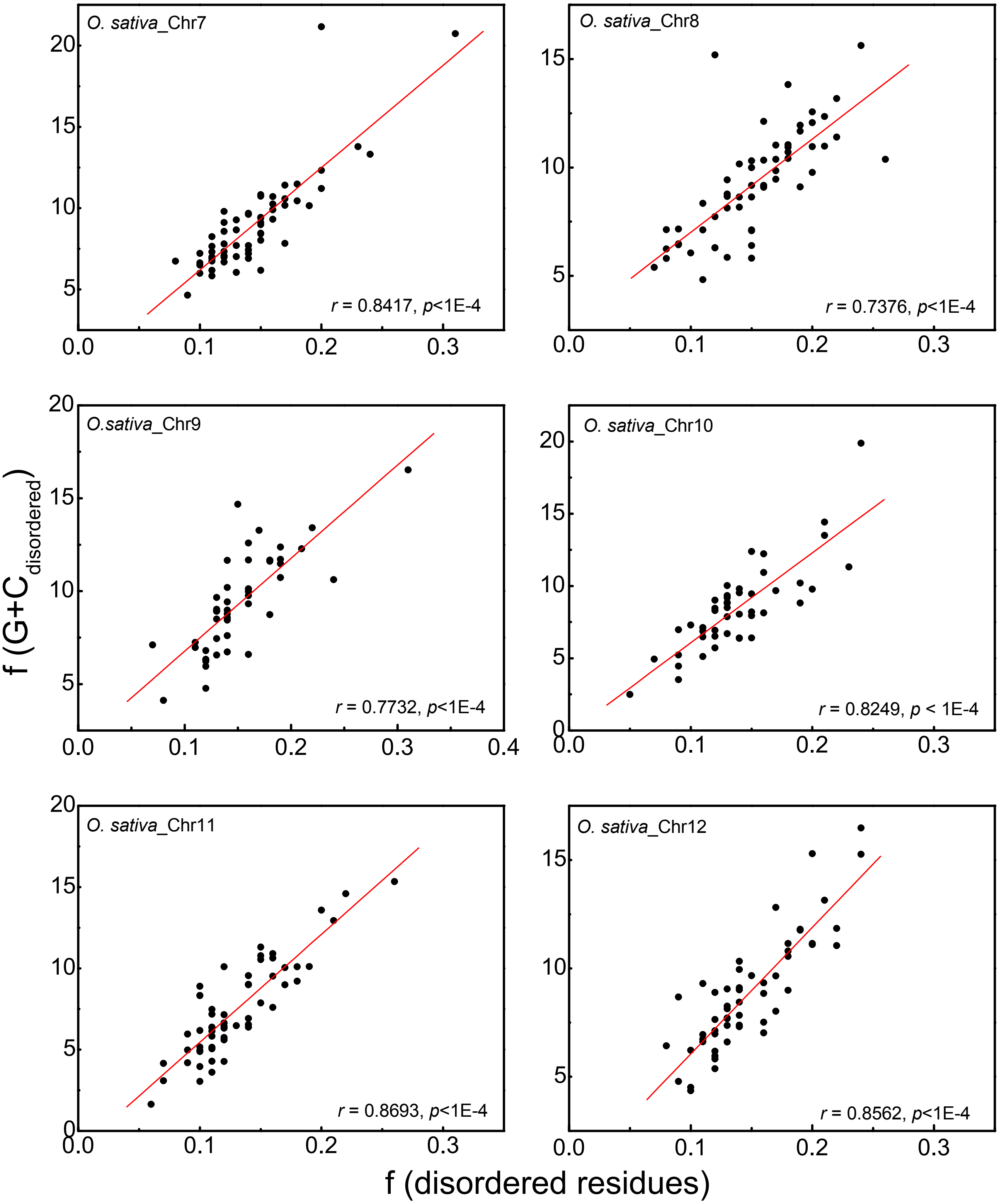
O. sativa_Chr7
r = 0.8417, p<1E-4
O. sativa_Chr8
r = 0.7376, p<1E-4
O.sativa_Chr9
r = 0.7732, p<1E-4
O. sativa_Chr10
r = 0.8249, p < 1E-4
O. sativa_Chr11
r = 0.8693, p<1E-4
O. sativa_Chr12
r = 0.8562, p<1E-4
f (G+C_disordered)
f (disordered residues)

Figure S2C

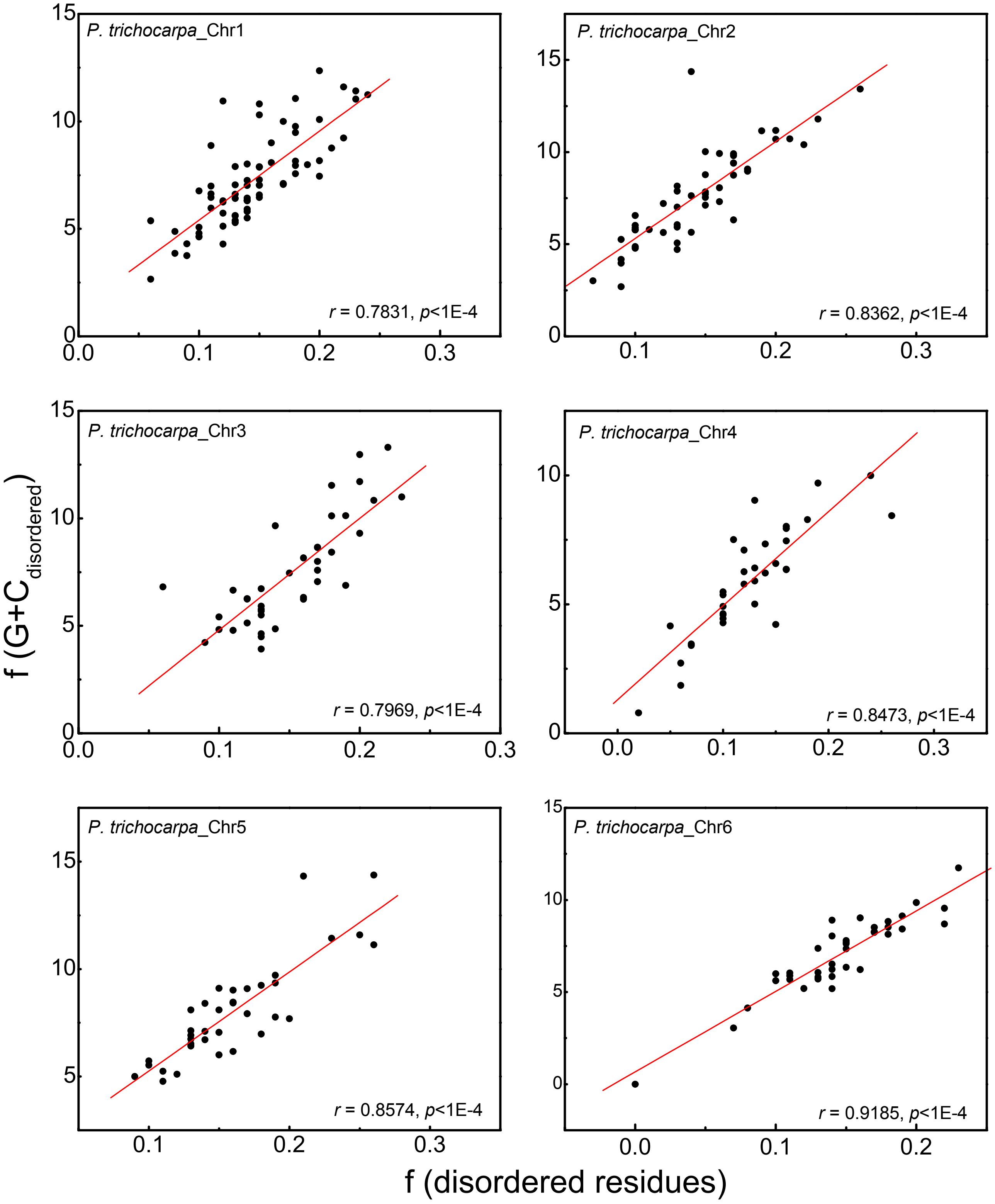
P. trichocarpa_Chr1
r = 0.7831, p<1E-4
P. trichocarpa_Chr2
r = 0.8362, p<1E-4
P. trichocarpa_Chr3
r = 0.7969, p<1E-4
P. trichocarpa_Chr4
r = 0.8473, p<1E-4
P. trichocarpa_Chr5
r = 0.8574, p<1E-4
P. trichocarpa_Chr6
r = 0.9185, p<1E-4
f (G+C disordered)
f (disordered residues)

Figure 2D

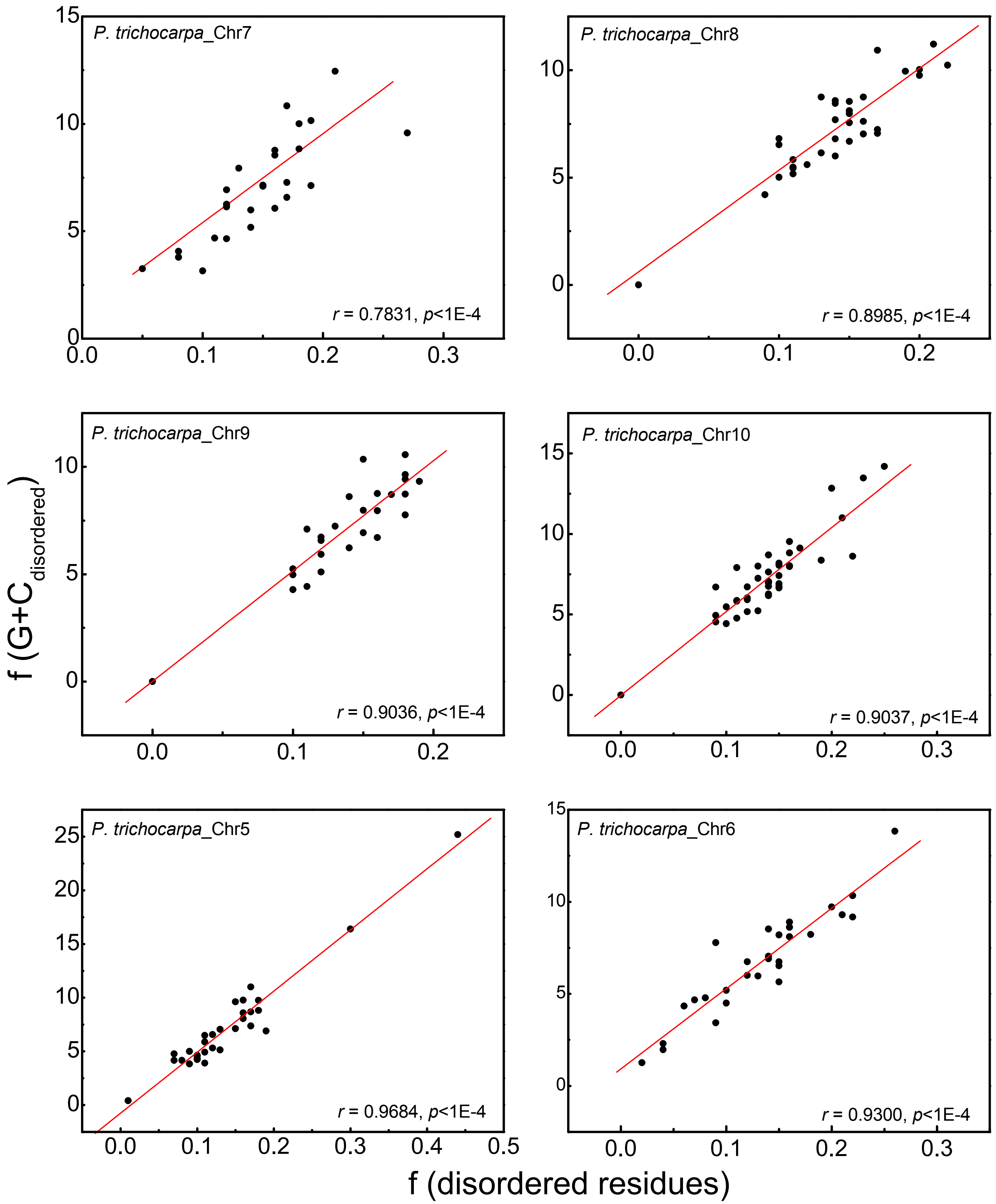
P. trichocarpa_Chr7
r = 0.7831, p<1E-4
P. trichocarpa_Chr8
r = 0.8985, p<1E-4
P. trichocarpa_Chr9
r = 0.9036, p<1E-4
P. trichocarpa_Chr10
r = 0.9037, p<1E-4
P. trichocarpa_Chr5
r = 0.9684, p<1E-4
P. trichocarpa_Chr6
r = 0.9300, p<1E-4
f (G+C disordered)
f (disordered residues)

Figure 2D

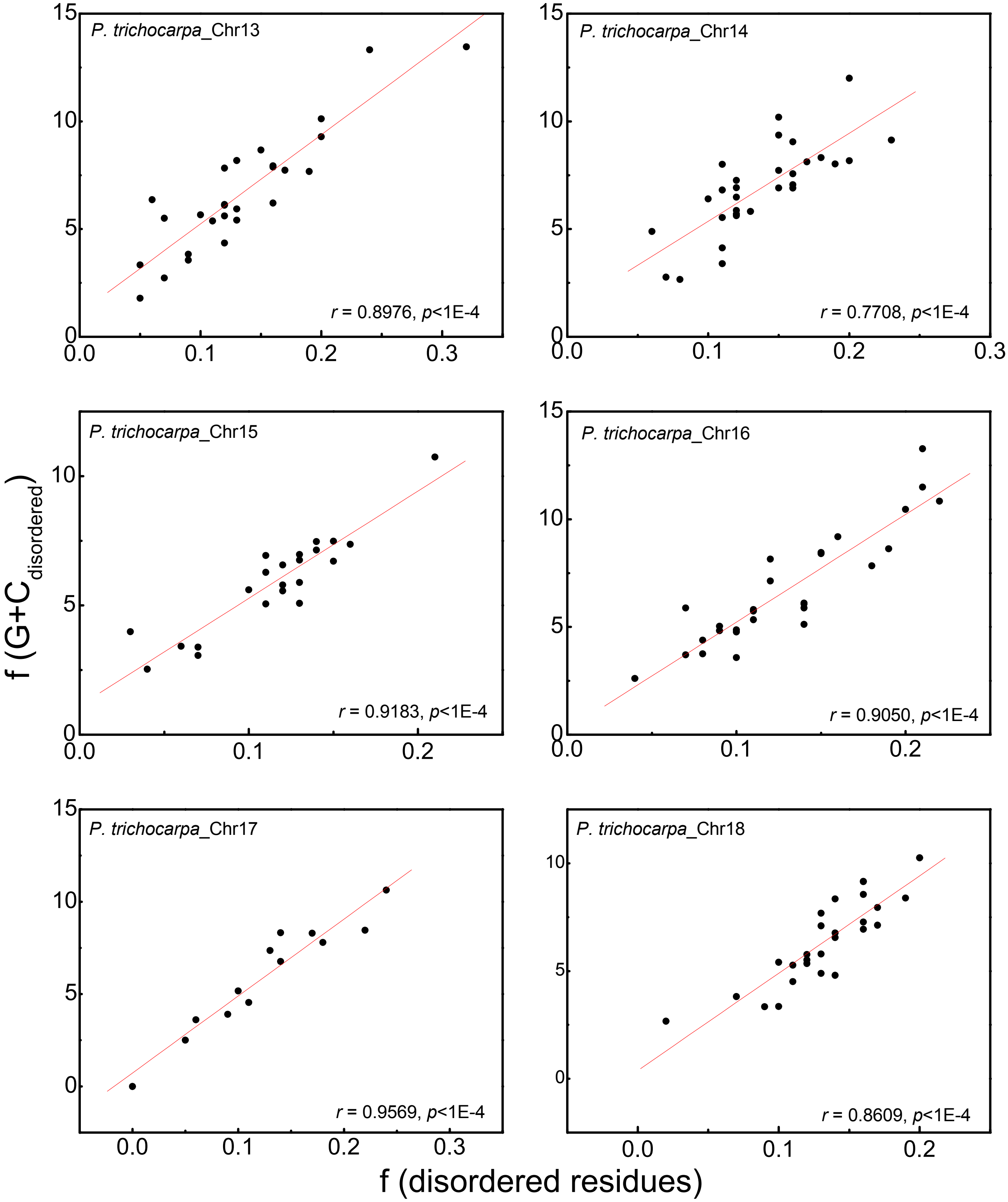
P. trichocarpa_Chr13
r = 0.8976, p<1E-4
P. trichocarpa_Chr14
r = 0.7708, p<1E-4
P. trichocarpa_Chr15
r = 0.9183, p<1E-4
P. trichocarpa_Chr16
r = 0.9050, p<1E-4
P. trichocarpa_Chr17
r = 0.9569, p<1E-4
P. trichocarpa_Chr18
r = 0.8609, p<1E-4
f (G+C disordered)
f (disordered residues)

Figure 2D

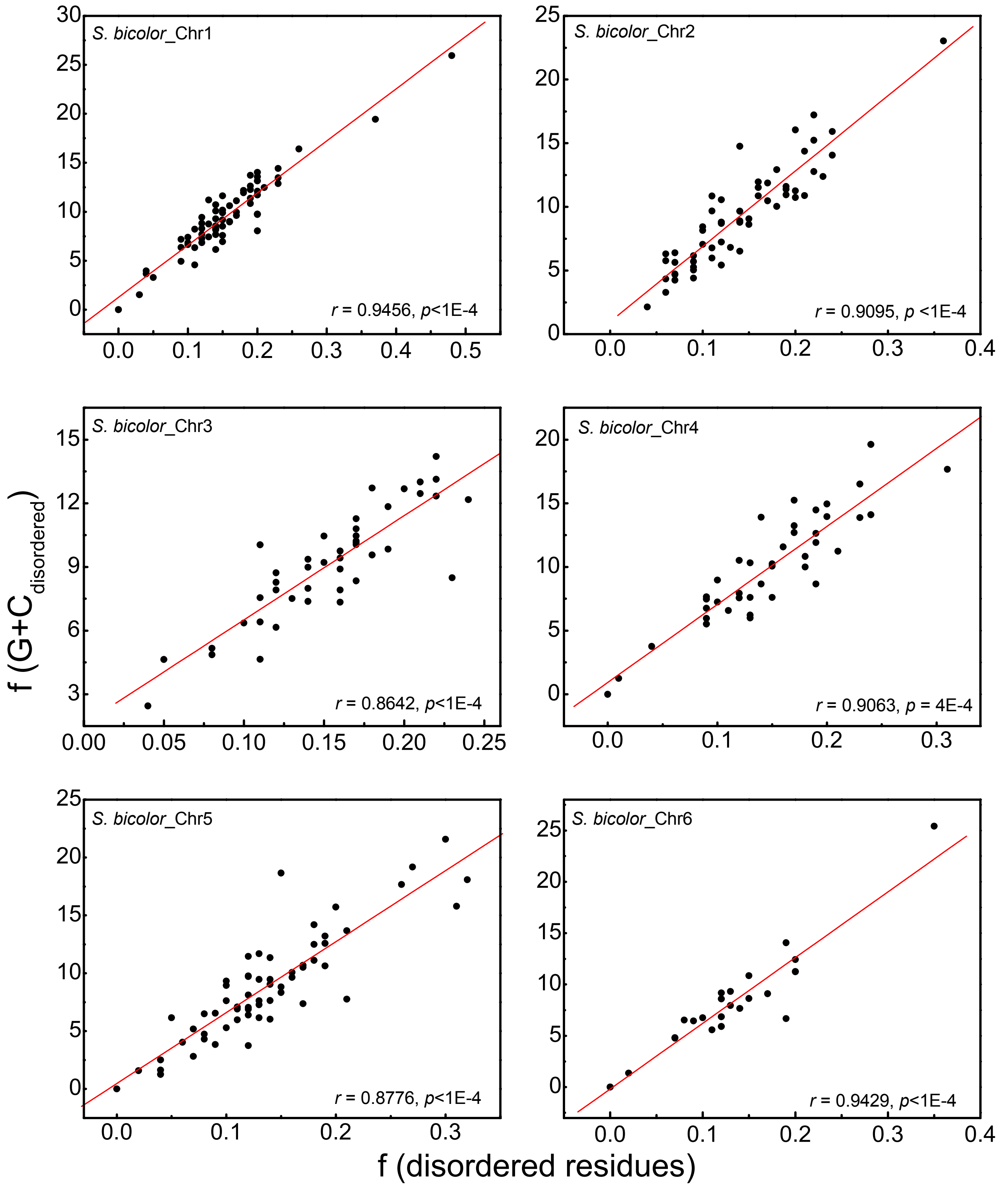

S. bicolor_Chr1
r = 0.9456, p<1E-4
S. bicolor_Chr2
r = 0.9095, p <1E-4
S. bicolor_Chr3
r = 0.8642, p<1E-4
S. bicolor_Chr4
r = 0.9063, p = 4E-4
S. bicolor_Chr5
r = 0.8776, p<1E-4
S. bicolor_Chr6
r = 0.9429, p<1E-4
f (G+C disordered)
f (disordered residues)


Figure 2E

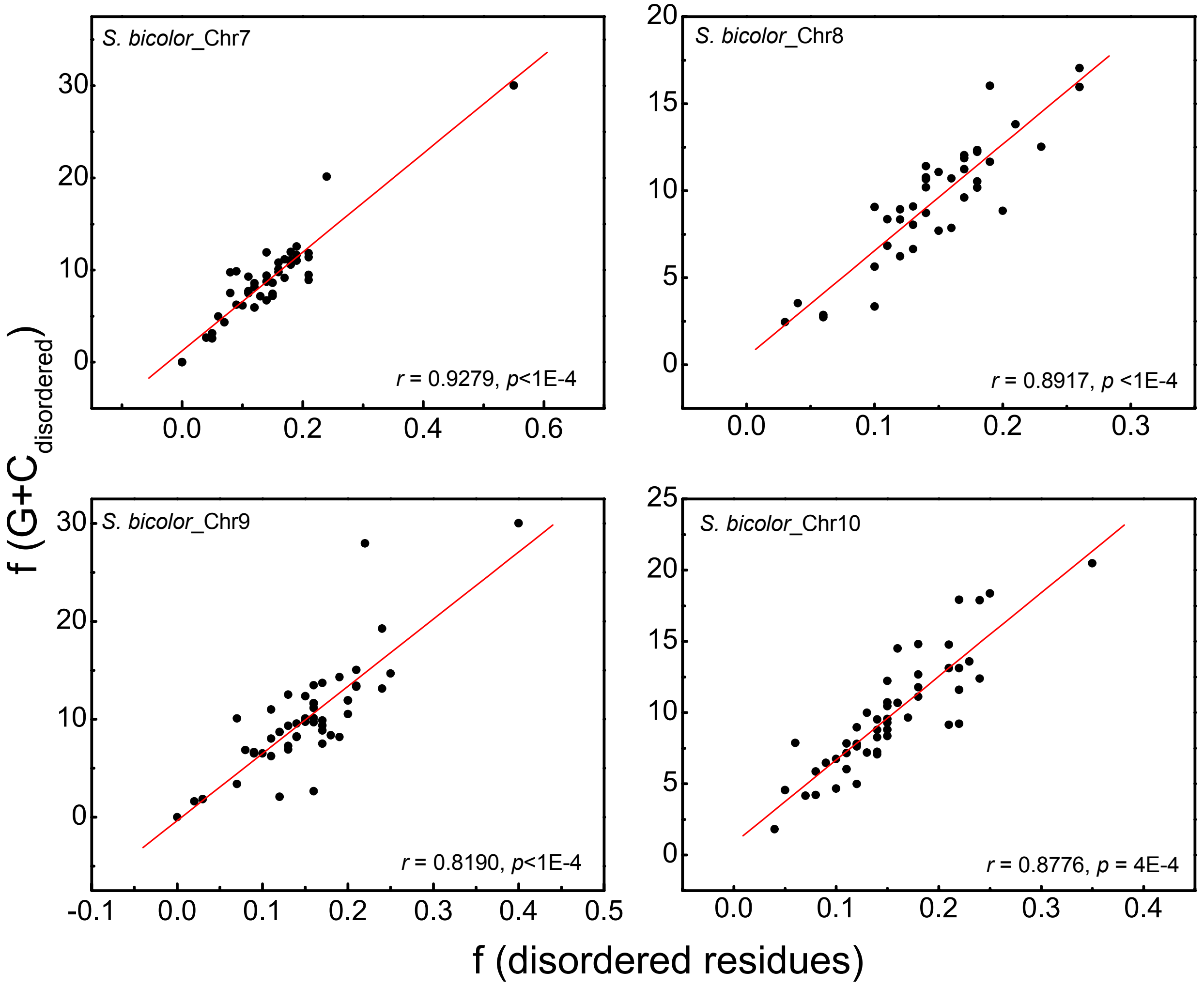
S. bicolor_Chr7
r = 0.9279, p<1E-4
S. bicolor_Chr8
r = 0.8917, p <1E-4
S. bicolor_Chr9
r = 0.8190, p<1E-4
S. bicolor_Chr10
r = 0.8776, p = 4E-4
f (G+C disordered)
f (disordered residues)

Figure 2E

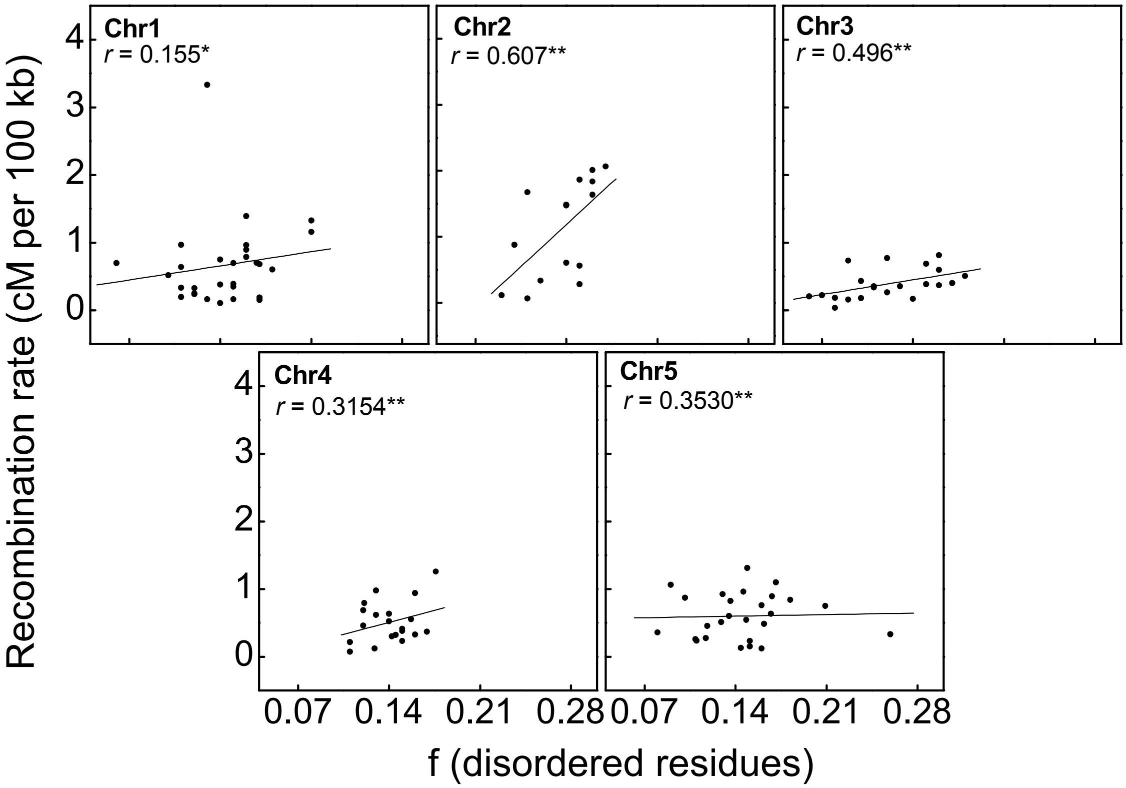

Chr1
r = 0.155*
Chr2
r = 0.607**
Chr3
r = 0.496**
Chr4
r = 0.3154**
Chr5
r = 0.3530**
Recombination rate (cM per 100 kb)
0
1
2
3
4
0.07
0.14
0.21
0.28
f (disordered residues)


Figure S3A

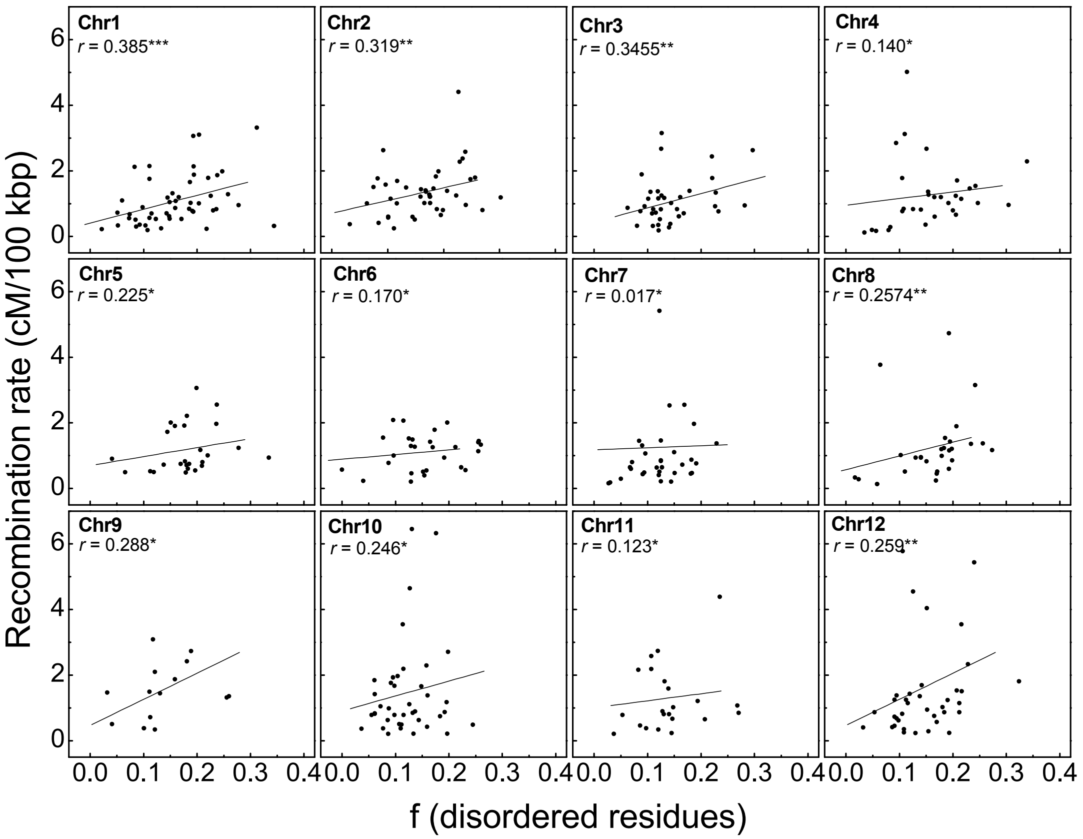

Recombination rate (cM/100 kbp)
f (disordered residues)
Chr1
r = 0.385***
Chr2
r = 0.319**
Chr3
r = 0.3455**
Chr4
r = 0.140*
Chr5
r = 0.225*
Chr6
r = 0.170*
Chr7
r = 0.017*
Chr8
r = 0.2574**
Chr9
r = 0.288*
Chr10
r = 0.246*
Chr11
r = 0.123*
Chr12
r = 0.259**


Figure S3B
